# Inside Honeybee Hives: Impact of Natural Propolis on the Ectoparasitic Mite *Varroa destructor* and Viruses

**DOI:** 10.3390/insects8010015

**Published:** 2017-02-06

**Authors:** Nora Drescher, Alexandra-Maria Klein, Peter Neumann, Orlando Yañez, Sara D. Leonhardt

**Affiliations:** 1 Institute of Ecology, Leuphana University of Lüneburg, Scharnhorststr. 1, Lüneburg D-21335, Germany; nora.drescher@gmx.de; 2 Department of Nature Conservation and Landscape Ecology, University of Freiburg, Tennenbacher Str. 4, Freiburg D-79106, Germany; alexandra.klein@nature.uni-freiburg.de; 3 Institute of Bee Health, Vetsuisse Faculty, University of Bern, Schwarzenburgstrasse 161, Bern CH-3003, Switzerland; peter.neumann@vetsuisse.unibe.ch (P.N.); orlando.yanez@vetsuisse.unibe.ch (O.Y.); 4 Swiss Bee Research Centre, Agroscope, Bern CH-3003, Switzerland; 5 Department of Animal Department of Ecology and Tropical Biology, University of Würzburg, Biocenter-Am Hubland, Würzburg D-97074, Germany

**Keywords:** *Apis mellifera*, deformed wing virus, plant-insect interactions, resin, sacbrood virus, social immunity

## Abstract

Social immunity is a key factor for honeybee health, including behavioral defense strategies such as the collective use of antimicrobial plant resins (propolis). While laboratory data repeatedly show significant propolis effects, field data are scarce, especially at the colony level. Here, we investigated whether propolis, as naturally deposited in the nests, can protect honeybees against ectoparasitic mites *Varroa destructor* and associated viruses, which are currently considered the most serious biological threat to European honeybee subspecies, *Apis mellifera*, globally. Propolis intake of 10 field colonies was manipulated by either reducing or adding freshly collected propolis. Mite infestations, titers of deformed wing virus (DWV) and sacbrood virus (SBV), resin intake, as well as colony strength were recorded monthly from July to September 2013. We additionally examined the effect of raw propolis volatiles on mite survival in laboratory assays. Our results showed no significant effects of adding or removing propolis on mite survival and infestation levels. However, in relation to *V. destructor*, DWV titers increased significantly less in colonies with added propolis than in propolis-removed colonies, whereas SBV titers were similar. Colonies with added propolis were also significantly stronger than propolis-removed colonies. These findings indicate that propolis may interfere with the dynamics of *V. destructor*-transmitted viruses, thereby further emphasizing the importance of propolis for honeybee health.

## 1. Introduction

Western honeybees, *Apis mellifera*, struggle with multiple environmental impacts, including numerous pests and pathogens. Particularly managed colonies of the European derived subspecies continue to suffer high (winter) losses, which is observed with increasing attention and concern [1,2,3,4]. Honeybees are effective pollinators of various crops, and managed honeybees become increasingly important in securing yields, particularly with decreasing diversity and abundance of many wild pollinator species [5,6] and with increasing demand for agricultural products [7,8].

The reasons for the observed elevated mortalities of managed honeybee colonies are still subject to debate [9,10,11,12,13,14,15]. Honeybees have been kept by humans for centuries, and it is likely that beekeeping practices (including breeding), which focus primarily on traits such as bee handling and productivity, conflict with the bees’ local adaptions [16,17,18]. Moreover, the commercial use and international trade of bees and bee products likely promotes the dispersal of diseases and pests [13,19,20,21]. Consequently, honeybees worldwide suffer from various pests and pathogens [21,22,23,24].

Among these, *Varroa destructor*, an ectoparasitic mite that feeds on the hemolymph of pupae and adult bees, is considered one of the most challenging threats [15,25,26,27,28]. Since these mites have shifted their host from the Eastern honeybee *A. cerana* to *A. mellifera*, *V. destructor* is spreading rapidly on almost every continent [3,23,25]. It has become a ubiquitous pest which commonly leads to colony collapse within two to three years, unless they are treated by beekeepers [25,29], because *V. destructor* vectors several honeybee viruses, which generate a fatal disease epidemic within the colony [11,30,31,32,33].

At least seven viruses have been detected in *V. destructor* mites (i.e., acute bee paralysis virus (ABPV), Kashmir bee virus (KBV), Israeli acute bee paralysis virus (IAPV), chronic bee paralysis virus (CBPV), sacbrood virus (SBV), deformed wing virus (DWV), and *Varroa destructor* virus-1 (VDV-1) [28,34,35,36], supporting its putative role in virus transmission. A particularly strong link is known for *V. destructor* and DWV. Several field and laboratory experiments demonstrated a positive correlation between mite infestations and virus titers [30,31,37,38,39,40]. Besides promoting horizontal transmission, these mites likely play a crucial role in activating latent virus infections [40,41]. While DWV is of generally low virulence and causes rather asymptomatic infections, it became one of the most prevalent viruses in apiaries and is highly pathogenic in association with *V. destructor* [11,32,35]. In contrast to DWV, other *V. destructor*-associated viruses, such as SBV, mostly occur less frequently and in rather moderate amounts in mites [35,42]. Unlike DWV, SBV titers are not directly correlated with *V. destructor* infestations, but are highest in spring and decrease towards late summer [35,43]. In contrast, DWV loads typically increase relative to *V. destructor* infestation from spring to late summer [30], thereby affecting the development of the long-living winter bees, which are essential for colony survival in temperate regions [26,30]. Thus, *V. destructor* and associated DWV can cause substantial damage to colonies and most likely play a key role for colony losses during winter [12,33,37].

Because dense aggregation of hosts in colonies can facilitate the spread of diseases, honeybees and other eusocial insects have evolved social immunity [44], including hygienic behavior (i.e., nest hygiene and allogrooming) [45,46,47], and social analogs of immune functions, such as social fever [48], encapsulation [49], and apoptosis [50] to combat pathogens. Honeybees [51], stingless bees [52,53], and some ant species [54,55] also collect plant resin, a sticky substrate secreted by plants to protect young sprouts and leaf buds [56], which is used for nest construction and defense against pests and pathogens (reviewed in [51]). Propolis, the apicultural term for a mixture from various plant resins and wax, has long been known for its antibiotic properties and been used by humans in traditional medicine for centuries (reviewed in [57,58]). Chemically, it is a complex and highly variable mixture with up to 300 different substances, whose composition depends on the available plant resources [59,60,61]. The functional properties of propolis derive mainly from a variety of water insoluble compounds, such as various phenolic constitutions (flavonoids, aromatic acids, and benzopyranes) and terpenoids [62,63,64,65,66,67]. Volatile substances generally represent only a small fraction (1%–3%) of the entire bouquet, but significantly contribute to typical propolis characteristics, such as its distinctive aroma and its biological activity [68,69]. The biological activity and pharmacological properties of propolis have been intensively investigated [60] with a wide range of antimicrobial [70,71], antifungal [72,73], antiviral [72,74], and immunomodulatory [75] properties described. However, while numerous studies examined the pharmacological value of propolis for humans, only few investigated how propolis benefits the bees themselves. For example, Simone-Finstrom et al. [76] showed that treating hives with propolis extract can reduce overall bacterial loads and immune activity in individual bees. Propolis was further found to be active against the causative agent of American foulbrood (*Paenibacillus larvae*) [77,78], as well as of chalkbrood (*Ascosphaera apis*) [79]. Propolis extracts can also have narcotic and lethal effects on *V. destructor* depending on the dosage used [80,81]. Consequently, propolis appears to be active against a range of honeybee pests and pathogens and can be considered a social immune defense mechanism, which is likely to be important for maintaining colony health [76,82,83,84]. However, the majority of studies which investigated the effect of propolis on honeybee pests used propolis ethanolic extracts, and therefore do not inevitably reflect the functional properties of propolis in its natural (solid) form as placed in hives by bees. Moreover, testing effects of propolis on certain pathogens in laboratory assays may not necessarily implicate colony level effects [85].

In this study, we therefore investigated the effect of raw propolis on four common honeybee viruses and on *V. destructor* mite infestations. We manipulated the amount of resin present within a hive by either removing or adding resin deposited above the brood nest. Due to the known antiviral properties of propolis [74,86,87,88], we hypothesized that viral titers would be reduced in colonies treated with extra propolis compared to propolis-removed colonies. We further expected that *V. destructor* infestation would be reduced due to the possible acaricide activity of propolis.

## 2. Material and Methods

### 2.1. Bioassay to Test for Effects of Propolis on Virus Infections and Mite Loads

#### 2.1.1. Experimental Site and Set-Up

The experimental site was located in an agricultural landscape close to Lüneburg, lower Saxony, Germany, comprising comparatively small arable fields with varying crops (such as rapeseed, grain, sugar beet, and potato) and pastures as well as some scattered trees and mixed forest patches. The experiment was conducted between June and October 2013 (local summer/early autumn). Ten honeybee colonies with young, naturally mated queens were established in May 2013 in clean propolis-free wooden Dadant boxes consisting of a single brood chamber. Experimental colonies where maintained by splitting colonies of a local stock of *Apis mellifera carnica*, which were all reared in the same apiary and provided with three brood frames as well as honey and pollen storage. Source colonies had routinely been treated against ectoparasitic mites *V. destructor* with organic acids in the previous winter (November 2012), and none of them showed any obvious clinical disease symptoms during visual inspections.

Experimental manipulations of propolis were conducted between June, when bees initiated substantial resin collection (noticed by the deposits in propolis traps and/or inner hive parts), and September 2013. All 10 hives were placed in pairs of two neighboring colonies (high and low propolis treatment) with a distance of 0.25 m and placed in a row with a distance of 1.5 m between the pairs. Five colonies were provided with additional propolis—in the following called “high” (propolis) treatment—that was removed from the other half—referred to as having “low” (propolis) treatment. A commercial plastic propolis trap (Logar trade d.o.o., Šencur, Slovenia) was placed on the top of the frames to stimulate resin deposition. We assumed that bees deposit a considerable amount of the gathered resin into the grids, but some resin was clearly also deposited elsewhere within the hive. To remove as much of the gathered resins as possible from the “low” treatment group, propolis grids were removed from the low propolis colonies once per week in June and every three days between July to September 2013. Grids were stored in a freezer and propolis deposits on grids were collected by flexing the frozen grids to break off propolis. Propolis from frames and inner hive walls was further removed by scratching it off with an apiary chisel. Following the collection, propolis was mixed, ground, weighed, and evenly distributed across the five “high” propolis treatment colonies by placing it on top of the hive frames no later than one day after removal from the “low” treatment colonies. Each portion of grounded propolis was compressed and gently pressed on the frames to ensure that propolis remains on the frames. Overall, each “high” propolis treatment colony received 16 g (± SD 0.5) of extra propolis, which represented between 9% and 70% (median 12%, ± SD 26) of the amount of propolis deposited in their own propolis grids.

#### 2.1.2. Data Collection and Sampling

Quantitative resin intake per colony was quantified by weighing all grids each time propolis was removed. As the amount of resin collected by the bees varied between colonies and often exceeded the amount of propolis which was used for the “high” propolis treatment, we additionally tested for possible correlations between pathogen loads and propolis collection (see below). The status of all colonies was controlled by estimating colony strength (see below) and verifying queen-state once per month between the end of June and the beginning of September 2013. Further, winter survival of all colonies was controlled in March 2014.

#### 2.1.3. Bee Sampling and Determination of Mite Infestation Levels

The sampling protocol for assessing virus [43] and mite infestation [89] followed the COLOSS guidelines for *A. mellifera* pest and pathogen research. Once per month (July, August, September), ~300 adult workers were sampled from three brood free frames located next to the brood of each colony [43], placed immediately on ice, and subsequently stored at −80° C until analyses. Phoretic mite infestation levels were investigated for each colony by the alcohol washing method described in Dietemann et al.[90] and by counting dead mites which had fallen on bottom boards (i.e., natural mite fall) (see Supplementary Material). Therefore, bottom boards were placed underneath each hive and covered with a thick (0.3 cm) layer of aroma free petrolatum, to prevent wind or predators (e.g., ants) from removing mites and thus biasing mite quantification (“sticky board method”) [90,91]. For moderate infestation levels, mite fall—i.e., natural mite mortality—is known to be directly correlated with mite population size in a colony and can therefore be used as a proxy for overall mite infestation [92]. Dead mites which had fallen on bottom boards were counted every 24 h over a time period of 10 days each month. Total numbers of mites were divided by the total number of days to calculate daily mite fall. Daily mite fall was further divided by the colony strength to estimate relative mite infestation rates for each colony. Thus, mite infestation is hitherto presented as infestation per 1000 bees. Mite infestation rates as calculated by both methods (alcohol washing and natural mite fall) were highly correlated (Kendall’s rank correlation: *z* = 3.05, *p* = 0.002), and we therefore used only natural mite mortality in the statistical analyses.

Parallel to bee sampling, colony strength was recorded as the number of adult bees and brood cells according to the “Liebefelder-method” [93]. We recorded colony size early (before sunrise) in the morning, before flight activity started. For each side of every frame within a hive, the proportion occupied by bees was visually assessed. Proportions were then summed up for all frames within a hive to obtain the total number of frames occupied by bees. We finally calculated the total number of bees for each hive by multiplying the number of frames fully occupied by bees with the number of bees, which fit onto one side of a frame (i.e., ~1400 bees per 1130 cm^2^ for Dadant hives).

Brood cells were quantified using a grid with 1.5 × 1 × 0.5 dm^2^ square fields to estimate the total area of brood on each side of a frame. The total brood area (in dm^2^) was then converted into brood cells by multiplying brood area with the average number of cells occupying 1 dm^2^ (i.e., 400 cells for Dadant hives).

#### 2.1.4. Determination of Viral Loads

Pooled samples of ~300 adult workers per colony were used for the detection and quantification of four common honeybee viruses: (1) black queen cell virus (BQCV); (2) deformed wing virus (DWV); (3) Israeli acute paralysis virus (IAPV); and (4) sacbrood virus (SBV). Virus analyses were performed using reverse transcription and quantitative PCR following [43,94]. In brief, each sample was homogenized in TN buffer (10 mM Tris—10 mM NaCl, pH 7.6) using MACS M tubes with a Dispomix^®^ Drive homogenizer (Medic tools, Zug, Switzerland). RNA was then extracted using the RNA II NucleoSpin kit (Macherey-Nagel, Dueren, Germany) and eluted in 50 µL of RNAse-free water following the manufacturer’s instructions. Reverse transcription was performed using the M-LV RT enzyme (Promega, Madison, WI, USA) with 1 µg of extracted RNA and 100 μM of random hexamers in a 25 µL final volume following the manufacturer’s recommendations. Viral loads were determined by a relative quantification method using, as standard curves, serial dilutions of purified PCR products of known concentration. The qPCR reaction for each sample was performed in duplicate using Kapa SYBR^®^ Fast Master Mix (Kapa Biosystems, Woburn, MA, USA). Individual reactions contained 10 µL master mix, 3 µL cDNA template, 0.4 µL forward and reverse target primers (10 mM; Table 1) and 6.2 µL of Milli-Q water. Reactions were performed in an EcoTM Real-Time PCR System (Illumina, San Diego, CA, USA) thermocycler. The following cycling conditions were used: 3 min at 95 °C, followed by 40 cycles of 95 °C for 3 s, and 55 °C for 30 s, during which fluorescence measurements were taken. A melting curve (95 °C for 15 s, 55 °C for 15 s, and 95 °C for 15 s) was performed at the end of each run to ascertain the amplification of the target.

The target gene (Cq) was quantified in relation to a non-regulated reference gene (β-actin analyzed in parallel in each sample). We calculated efficiency corrected ratio values according to Pfaff to account for differences in PCR efficiency [98]: $Ratio:\;\frac{E_{target}Ʌ-Cq\;Virus\left[sample\right]}{E_{ref}Ʌ-Cq\;Actin\left[sample\right]}$, with E_target_ = real time PCR efficiency of target gene transcript, E_ref_ = real time PCR efficiency of reference gene transcript and Cq = quantification cycles.

### 2.2. Bioassay to Test for Effect of Propolis on V. destructor Mite Survival

Whether raw propolis affected the survival of adult *V. destructor* mites, was tested in a laboratory assay, in June 2014 and again in August 2014. Propolis for the assay was obtained from two honeybee colonies near Lüneburg, Lower Saxony, Germany between June and August 2013, using a commercial propolis grid (see above). In order to test for a cofounding effect of a thymol based acaricide treatment, one colony had previously been treated with thymol (propolis B), whereas the other one had not received any thymol treatment (propolis A). Propolis collected from each colony was grounded separately using an electric coffee mill (typ3871, Severin, Sundern, Germany) and stored at −18 °C. *V. destructor* mites were collected prior to the experiment from infested brood of three colonies placed in the same apiary.

#### Collection of *V. destructor* Mites and Performance of Bioassays

In order to obtain mites, sealed brood was removed from brood combs and transferred to the laboratory where we opened the wax cap of each brood cell, removed pupae and collected attendant mites with forceps. Only the larger female mites with dark color were used for the assays, while those that appeared to have recently malted (pale color) or did not move were discarded. Prior to testing, mites were kept in petri dishes together with a bee pupa as described in Delaplane et al. [93] to prevent starvation.

For bioassays, 6 g of grounded propolis were placed in the lid of a plastic container (160 mL). Between 10 and 14 mites were placed in the bottom of each container with a small piece of moist tissue. Propolis and mites were separated by a nylon net which prevented direct contact, but allowed free evaporation of volatiles. A container without any propolis was used as control. We performed five repeats for each propolis sample and the control. Fresh mites were used for all tests. All containers were kept in an incubator at 34 °C for 36 h. Following the protocol of Garedew et al. [80] and Damiani et al. [99], mite survival was monitored under a microscope 12, 18, 24, and 36 h after the assay had started. Mites were classified as “mobile” when they were still active (i.e., able to move extremities); “immobile” when they were inactive, but still alive (as validated by careful touching them with a needle); or “dead” when they showed no movement after three subsequent needle stimulations.

### 2.3. Statistical Analyses

Generalized linear mixed effect models (GLMM, glmer function in lme4 package) were used to analyze the effect of propolis treatment on honeybee worker viral loads (normalized fold ratios). Data of viral loads were log2-transformed to achieve normality. For the SBV load, only data from August and September were used, as SBV titers peaked before, or approximately with the beginning of the experiment, which may interfere with possible treatment effects. Due to the known positive correlation between *V. destructor* infestation and the degree of viral infection as described in Shen et al. [40], Dainat et al. [30] and Francis et al. [31], differences in *V. destructor* infestation between colonies may have masked treatment effects. Therefore, initial models for each virus included “treatment”, “*V. destructor* infestation rate”, and their interaction as explanatory variables. To account for possible colony specific differences in initial viral loads and viral fluctuations over the season, “colony” and “sampling time” were included as random factors in all models. We used backward elimination and maximum likelihood ratio estimators to fit the most parsimonious model. Models were ranked based on their Akaike’s information criterion (AIC) values and compared to the null model (including only the random variables colony and sampling time) using likelihood ratio tests (ANOVA command in the lme4 package). We additionally compared the ratio of viral titers and mite infestation (ratio = virus titer/*V. destructor* mite infestation) between the two treatment groups using Wilcoxon rank sum tests to confirm GLMM results. Ratios were calculated for each colony and each sampling point.

We further tested for a possible correlation between quantitative resin collection and pathogen loads (*V. destructor* mite, viral infection) using Kendall’s rank correlation tests. Correlations were tested for all colonies pooled as well as for each treatment group separately.

We composed and compared additional GLMMs to examine whether *V. destructor* infestation, virus loads, or colony size best explained the amount of resin collected by colonies. Here, initial models included the explanatory variables treatment and either *V. destructor* infestation rate, or viral load, or the number of adult bees. Models were fitted as described above with “colony” and “sampling time” included as random factors in all models, and the variances explained by each model were compared by marginal (R^2^m) and conditional R (R^2^c) squared values following Nakagawa [50] (Multi-model inference, r.squaredGLMM function in MuMIn package). A Wilcoxon rank sum test was further used to compare quantitative resin collection between the two treatment groups.

In order to assess the survival of *V. destructor* mites exposed to propolis and to test for differences between propolis samples and the control, the Kaplan Meier estimator (corrected for multiple testing with Bonferroni) was used (surv function in survival package). For survival analyses, we combined data for the categories “immobile” and “dead” as both would lead to dropping of mites from bees and thus prevent them from causing further harm to bees. Therefore, numbers of immobile and dead mites were summed up and compared to the number of mobile mites for each record. All statistical analyses were performed in R [100].

## 3. Results

### 3.1. Resin Intake and Colony Strength

**Figure 1:**
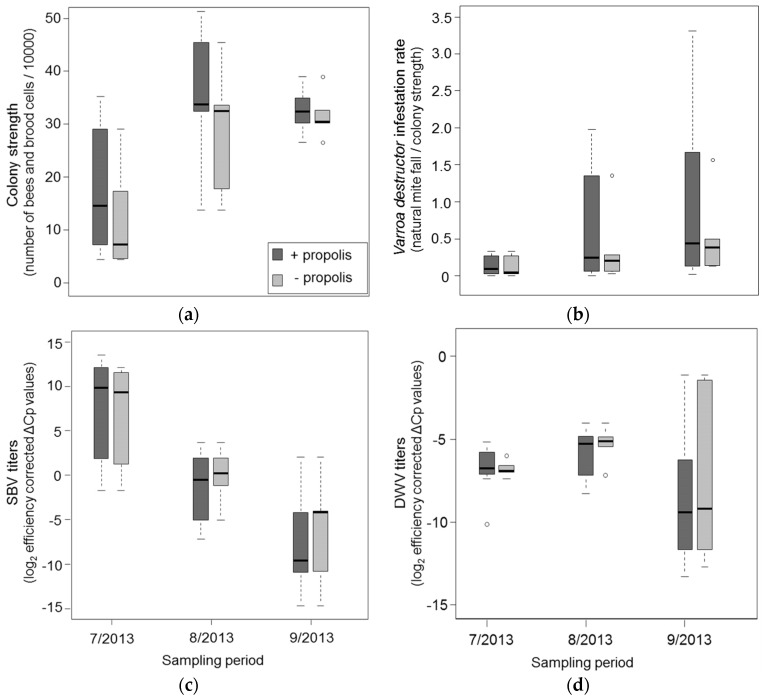
(**a**–**d**) Effects of the high (dark grey) and low (light grey) propolis treatment on the development of (**a**) colony strength; (**b**) *Varroa destructor* infestation rates (natural mite fall/colony strength); (**c**) sacbrood virus titers (SBV); and (**d**) deformed wing virus titers (DWV) over the course of the experiment from July to September 2013. Viral titers are expressed as log_2_ transformed, efficiency corrected ΔCq values (Cq = quantification cycles). Each boxplot represents median values of both treatment groups (N = 5) per month with default ranges for boxes (75th and 25th percentile), whiskers (±1.5) and outliers (dots).

After bees initiated resin intake in June, it was low for the first 20 days (mean amount of resin ± SD: 0.9 g/colony and day ±0.5) and increased from the second half of July to August (2.8 g/colony and day ±1.9). Resin intake decreased again from late August (0.75 g/colony and day ±0.2) to September (0.6 g/colony and day ± 0.3). The total amount of resin collected over the experimental period ranged from 28 g to 205 g (mean ± SD: 131.2 g ± 71.3) per colony and was positively correlated with colony strength (Kendall’s rank correlation: *z* = 3.52, *r* = 0.46, *p* < 0.001). Colony strength was further higher in colonies which received additional propolis (Wilcoxon rank sum test: *W* = 63, *p* = 0.041, Figure 1a).

### 3.2. Effects of Propolis Manipulation on Virus Infections and Mite Loads

*Varroa destructor* infestation generally increased from June to September 2013 in all experimental colonies (Figure 1b), but mite infestation levels strongly varied among colonies (Figure 1b) and were higher in stronger colonies (Kendall’s rank correlation: *z* = 3.43, *p* = 0.001). We found no significant effect of propolis treatment on mite infestation (Wilcoxon rank sum test: *W* = 97, *p* = 0.534). Of the four viruses assessed in the study, only SBV and DWV were detected. Viral titers differed between the colonies, but generally increased (DWV, Figure 1d) or decreased (SBV, Figure 1c) from July to August. Increase of DWV was interrupted after, at the beginning of September, all colonies received a thymol based acaricide treatment.

**Figure 2:**
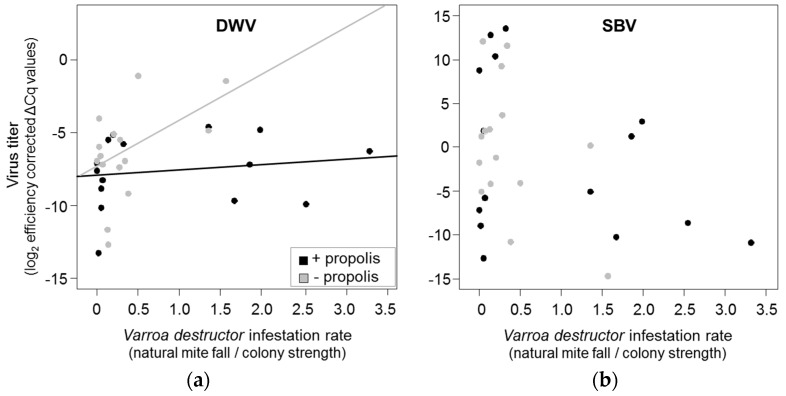
(**a**,**b**) Effect of the high (black) and low propolis (grey) treatment on the correlation between viral loads and the V. *destructor* mite infestations of (**a**) deformed wing virus (DWV); and (**b**) sacbrood virus (SBV). Each dot represents data from one colony for one month. Virus titers and *V. destructor* infestation were measured for each colony once per month from July to September 2013. Lines represent linear regressions between DWV virus titers (log_2_ efficiency corrected fold-change relative to housekeeping gene, Cq = quantification cycles) and *V. destructor* infestation rates (natural mite fall/colony strength) for each treatment group according to significant interaction between treatment and *V. destructor* infestation (see Table 2).

Overall, DWV titers were best explained by an interaction between propolis treatment and *V. destructor* infestation (Table 2). In colonies that received additional propolis, DWV titers increased less in relation to *V. destructor* infestation than in propolis-removed colonies, and the ratio of DWV and *V. destructor* was significantly lower (Wilcoxon rank sum test: *W* = 141, *p* = 0.046, Figure 2a). In contrast, none of our variables explained a significant proportion of the variance in SBV titers (Table 2), and the ratio between SBV and *V. destructor* did not differ between treatments (Wilcoxon rank sum test: *W* = 56, *p* = 0.113, Figure 2b). All colonies survived the winter.

### 3.3. Correlation between Resin Intake and Pathogen Loads

**Figure 3:**
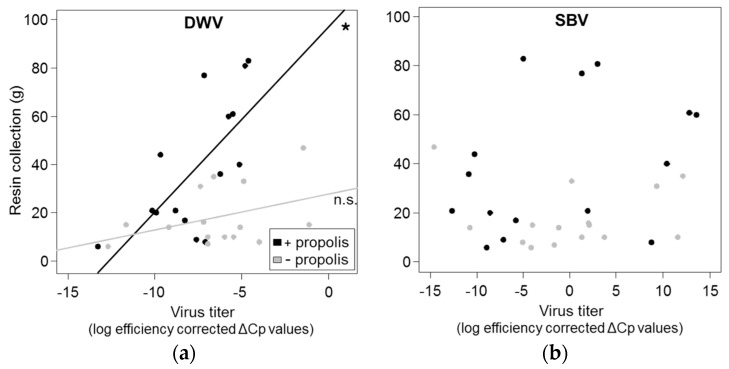
(**a**,**b**) Effect of viral infection with (**a**) deformed wing virus (DWV); and (**b**) sacbrood virus (SBV) on the amount of resin (g) collected by bees. Each dot represents data from one colony for one month. Lines represent linear regression between resin collection and virus titers (log efficiency corrected fold-change relative to housekeeping gene) for each treatment group (black = ”high propolis treatment”, gray = “low propolis treatment”) with * indicating a significant correlation with *p* < 0.05 and n.s. a non-significant correlation.

Resin intake was generally higher in colonies with added propolis than in propolis-removed colonies (Wilcoxon rank sum test: *W* = 58, *p* = 0.025), and positively correlated with *V. destructor* infestation (Kendall’s rank correlation: *z* = 3.43, *r* = 0.45, *p* < 0.001), DWV titers (Kendall’s rank correlation: *z* = 1.95, *r* = 0.25, *p* = 0.026, Figure 3a) and colony strength (Kendall’s rank correlation: *z* = 2.34, *r* = 0.30, *p* = 0.01), but not with SBV titers (Kendall’s rank correlation: *z* = 0.70 *r* = 0.09, *p* = 0.243, Figure 3b). However, variance in the amount of resin collected by bees was best explained by the interaction of DWV and treatment (R^2^m = 32%, R^2^c = 72%, Figure 3a, Table 3). The positive correlations between resin collection and pathogen loads were highly significant for colonies with additional propolis (Kendall’s rank correlation: *V. destructor*: *z* = 2.43, *r* = 0.47, *p* = 0.008; DWV: *z* = 2.58, *r* = 0.5, *p* = 0.005), but weaker (Kendall’s rank correlation: *V. destructor*: *z* = 1.89, *r* = 0.37, *p* = 0.029) or not significant (Kendall’s rank correlation: DWV: *z* = 1, *r* = 0.20, *p* = 0.16) for propolis removed colonies (Figure 3a). No significant correlations were found for colony strength and resin collection, when treatment groups were tested separately (Kendall’s rank correlation: low treatment: *z* = 0.90, *r* = 0.18, *p* = 0.190; high treatment: *z* = 1.49, *r* = 0.29, *p* = 0.069).

### 3.4. Effect of Propolis on V. destructor Mite Survival

**Figure 4:**
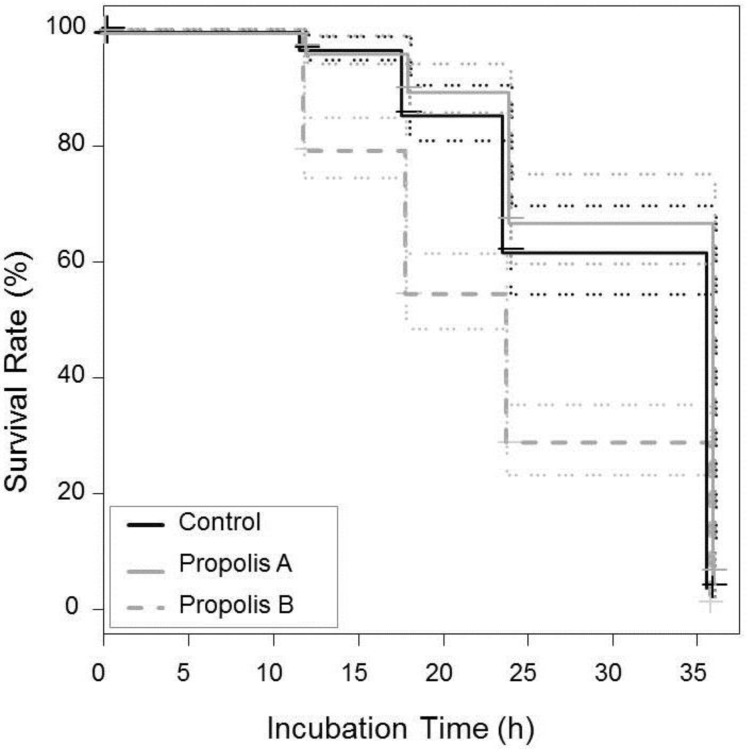
Kaplan-Meyer survival curves showing survival rates of *Varroa destructor* mites when exposed to propolis obtained from colonies treated with thymol (Propolis B = dashed line N = 58), not treated with thymol (Propolis A = gray line, N = 58) or not exposed to propolis (Control = black line, N = 58) under laboratory conditions. Dotted lines mark 95% confidence intervals.

Survival of *V. destructor* mites differed between propolis samples and the control (χ*^2^* = 103, *p* < 0.0001; Figure 4). Mites that were exposed to volatiles of propolis not treated with thymol (propolis A) lived about as long as the control group which had not been exposed to propolis (survival test: χ*^2^* = 1.5, *p* = 0.23; propolis A: median survival time ± SE = 36 h ± 0.03 h, control: 36 h ± 0.02 h; Figure 4). In contrast, propolis obtained from colonies treated with thymol significantly reduced mite survival (24 h ± 0.03 h) when compared with propolis A (χ*^2^* = 75.9, *p* < 0.0001) and the control (χ*^2^* = 59.2, *p <* 0.0001; Figure 4). After 36 h, on average, 96% of all mites had died irrespective of the treatment.

## 4. Discussion

Our results show that natural propolis can reduce DWV viral loads in relation to infestations with *V. destructor*, which further supports the key role of social immunity for eusocial insect health [101,102].

### 4.1. Effects of Propolis on Virus Infections and Mite Loads

Both viruses detected in our study, i.e., the deformed wing virus (DWV) and the sacbrood virus (SBV), are likely to be transmitted by *V. destructor* [35,103], but only DWV is known to be highly correlated with mite loads [30,31] and therefore usually increases from early summer to autumn [35,38]. Accordingly, DWV titers varied proportionally with *V. destructor* infestation in all of our colonies, whereas SBV titers decreased continuously from June to September, irrespective of mite infestation. Such seasonal dynamics observed for SBV confirm earlier studies (e.g., [35]). However, in colonies provided with additional propolis, DWV titers increased significantly less with *V. destructor* infestation compared to propolis-removed colonies. This finding suggests that the long known antiviral effect of propolis [74,86,87,88] may also account at the level of the honeybee colony. However, the effect of added propolis on DWV titers was relatively low and depended on *V. destructor* mite infestation. Thus, alternatively, but not mutually exclusive, bees in colonies with higher amounts of propolis may better cope with DWV infections due to a positive effect of propolis on their immune system [84]. Such a positive effect on colony health is further supported by significantly higher colony strength in colonies with added propolis in our study. Likewise, Nicodemo et al. [82] found increased brood viability and higher worker longevity in Africanized honeybees that were bred for high propolis production. Propolis may thus benefit honeybee colonies by increasing general vitality [76,79]. This increase in vitality may also explain why colonies that were provided with multiple grids to encourage formation of a “natural propolis envelope” were more likely to survive winter and were stronger in spring than colonies without envelope in the study of Borba et al. [84]. Our own study did not see an effect of propolis enrichment on survival over winter, but this may be due to the smaller sample size of six colonies (compared to twelve in Borba et al. [84]). Unlike our study, Borba et al. [84] found no difference in DWV infection between colonies with “natural propolis envelope” and colonies without. This discrepancy may be explained by differences in overall propolis amounts and thus DWV infection dynamics between studies. Moreover, Borba et al. [84] did not actively manipulate propolis amounts, quantify resin intake of colonies, or analyze the interaction between mites and DWV, which renders a direct comparison between studies difficult.

In contrast to DWV, we found no significant effect of propolis on SBV infection. This finding may be explained by the fact that SBV infection peaked approximately with the beginning of our experiment and then decreased in all colonies. Thus, differences in SBV infection between treatments may have been masked by the virus’ general demise.

Although antiviral activity of various propolis extracts against envelope and non-envelope viruses was demonstrated by several studies, the exact mode of action is still not completely understood [74,87]. Reduction of viral replication tends to be highest when extracts were applied prior to or at the time of infection, suggesting that propolis-derived compounds interfere with the binding or entering of viruses at host cells [87,88]. However, propolis is a complex mixture of various substances with a broad therapeutic spectrum, including immunomodulatory activity resulting primarily from stimulation of macrophage activity [87,88,96]. It was further shown to enhance the recognition of pathogens and support the initial steps of the immune response by upregulating the expression of two toll-like receptors [104]. In honeybees, viral defense is mediated mainly by RNA interference (RNAi), but also by immune-related mechanisms (i.e., the pathogen-associated molecular pattern (PAMP) and induced toll pathways) [105]. Thus, resistance to viral infections may be improved by propolis compounds through enhancing the non-specific immune defense, while simultaneously reducing the general investment in the cost intensive activation of humoral immune responses. In fact, the presence of propolis within bee hives was found to reduce the expression of immune-related genes without affecting levels of pathogens and parasites [84]. Other studies showed however that resin/propolis further reduced bacterial loads in the nests [54,76].

Constant removal of propolis may in turn increase physiological stress, which results in increased immune activity and thus reduced individual fitness. Such immune system weakening effects can play a critical role for viral infections such as with DWV [38,41,95]. While bees are generally able to combat DWV infections, physiological stress through mite pressure can enhance virus expansion and lead to a destabilized parasite-host relationship [27,32]. In fact, Nazzi et al. [32] found 19 immune related genes to be downregulated in *V. destructor*-infested colonies, indicating that mites not only function as vector for viruses, but also enhance viral infections by actively suppressing the immune function of bees. This finding was further confirmed by Ryabov et al. [106] who revealed that mite pressure increased replication of a virulent DWV strain, which was itself not detected in mites.

Our results suggest that the immune suppressive effect caused by mites may be (partly) compensated by the immune supportive effect of propolis, which can have important implications for colony health, as the levels of viral titers are known to be critical for the development of clinical disease symptoms [41]. Contrary to our expectations, our results showed no impact of propolis on *V. destructor* itself, which agrees with previous studies investigating the efficacy of natural propolis [37,83]. Survival of mites, in our study, was only reduced by raw propolis obtained from colonies that have previously been treated with thymol which is known to accumulate in propolis [107].

In contrast, other studies showed that direct exposure of mites to ethanolic propolis extracts can cause mortality and narcotic effects [80,99], suggesting that propolis can have a detrimental influence on mites. However, lethal effects were mainly observed for extracts with high alcohol content (70%) and less (or not) for extracts with lower alcohol content (40%), which may be explained by a comparable lower content of bioactive compounds [80]. Under natural conditions, mites rarely get in direct contact with high concentrations of non-volatile propolis compounds as found in 70% alcohol extracts [80], but may be exposed to volatiles evaporating from propolis deposited in varying areas of the hive (including cell rims). Our results thus indicate that such propolis volatiles do not affect mite survival (at least not for the propolis samples from our study area).

However, the chemical composition of propolis is highly variable and depends largely on the plant sources used by bees [59,108]. Bees may actually learn or evolve to shift their resin collection towards sources with acaricide activity as suggested by the study of Popova et al. [109] who found a higher content of three biologically active compounds in propolis from *V. destructor* resistant colonies (for a review see [110]). In fact, the existence of several *V. destructor* mite-surviving *A. mellifera* populations indicates that natural selection can lead to a stable parasite-host relationship, as seen, e.g., in African and Africanized honeybees which occur in large wild *V. destructor* mite resistant populations [97]. Compared to the European subspecies, African and Africanized honeybees generally collect larger amounts of propolis and have a better resistance against most of the common honeybee diseases [111], which further supports the beneficial role of propolis for *A. mellifera* colony health.

### 4.2.Correlation between Resin Intake and Pathogen Loads

Resin intake was comparatively higher in colonies with added propolis which may be explained by the observed differences in colony size between the two treatment groups. Interestingly, the quantity of resin collected by each colony was, overall, positively correlated with levels of DWV infection, suggesting that bees actively respond to viral pressure by increasing resin collection. Such “self-medication” was also described by Simone-Finstrom and Spivak [79], who showed that colonies increased resin foraging rates after having been challenged with the fungal parasite *Ascosphaera apis* (causative agent of chalkbrood).

However, we observed no such correlation between resin collection and SBV titers. As the collection of resins depends on its availability, which increases towards the end of summer [56], the apparent lack of response towards SBV infection may also be related to the earlier peak of SBV infection.

## 5. Conclusions

In conclusion, our study provides new insights into the functional properties of propolis as a colony level defense mechanism and thereby further supports its substantial role for honeybee colony health. We have shown that propolis can naturally benefit honeybee field colonies by reducing DWV loads in relation to *V. destructor* infestation, suggesting that it may be vital for bees to overcome this pathogen challenge. However, future studies (ideally with more colonies) should verify this interaction and follow colony strength, health, and viral titers over winter to better understand whether and (if so) how reduced DWV loads actually impact colony survival. Further studies including additional health parameters are also needed in order to better understand the actual mode of action of propolis. Our result on the activity of propolis against *V. destructor* contradicts previous studies which used ethanolic extracts and stresses the general need for more studies conducted under natural conditions at the colony level to obtain more biologically relevant data [85]. We further suggest that missing resin sources or the removal of propolis may have negative implications for honeybee colonies. Resin collection, as a natural defense of honeybees, should therefore find more consideration in practical beekeeping, particularly as many bee keepers constantly remove propolis from bee hives during routine control.

## Figures and Tables

**Table 1:** Primers, sequences, and references used for quantification of the honeybee viruses black queen cell virus (BQCV), deformed wing virus (DWV), Israeli acute paralysis virus (IAPV), and sacbrood virus (SBV). Target virus genes were quantified in relation to a non-regulated *Apis mellifera* (*A. m.*) reference gene (β-actin analyzed in parallel in each sample).

Assay	Primers	Sequence (5′–3′)	Reference
BQCV	BQCV-qF7893	AGTGGCGGAGATGTATGC	Locke et al. [95]
	BQCV-qB8150	GGAGGTGAAGTGGCTATATC	
DWV	DWV-F8668	TTCATTAAAGCCACCTGGAACATC	Forsgren et al. [96]
	DWV-B8757	TTTCCTCATTAACTGTGTCGTTGA	
IAPV	IAPV-F6627	CCATGCCTGGCGATTCAC	de Miranda et al. [97]
	KIABPV-B6707	CTGAATAATACTGTGCGTATC	
SBV	SBV-qF3164	TTGGAACTACGCATTCTCTG	Locke et al. [95]
	SBV-qB3461	GCTCTAACCTCGCATCAAC	
β-actin (*A.m.*)	Am-actin2-qF	CGTGCCGATAGTATTCTTG	Locke et al. [95]
	Am-actin2-qB	CTTCGTCACCAACATAGG	

**Table 2:** Results of different mixed-effects models testing for effects of high and low propolis treatment (“Treatment”) and *V. destructor* infestation (“Varroa”) with (Treatment × Varroa) and without (Treatment + Varroa) interactions included (=explanatory variables) on titers of the deformed wing virus (DWV) and the sacbrood virus (SBV) (=response variables) over three months (number of samples N = 30, three repeats per colony). In all models, colony and month were included as random factors to take into account colony-specific differences and repeatedly measuring the same colony. Table shows degrees of freedom (df) and p-values (*p*) for comparing all models presented against the null model (i.e., a model including only colony and sampling date as random factors). Significant *p*-value is marked in bold.

Response Variables	Explanatory Variables	df	*p*
DWV	Treatment × Varroa	7	**0.027**
	Treatment + Varroa	6	0.108
	Treatment	5	0.263
	Varroa	5	0.282
SBV	Treatment × Varroa	7	0.065
	Treatment + Varroa	6	0.219
	Treatment	5	0.086
	Varroa	5	0.623

**Table 3:** Results of mixed-effects models testing for effects of *V. destructor* infestation (“Varroa”), DWV infection (DWV) and treatment (“Treatment”) as well as their interactions (indicated with x) on the quantity of resin collected by each colony over three months (number of samples N = 30, three repeats per colony). In all models, colony and month were included as random factors to take into account colony-specific differences and repeatedly measuring the same colony. Table shows *p*-values (*p*) and *R^2^*-values (i.e., percentage explained variance) for comparing all models presented against the null model (i.e., a model including only colony and sampling date as random factors), marginal R squared values (R^2^m: representing the explanatory power of the fixed effects only) and conditional R squared values (R^2^c: representing the explanatory power of the whole model, including the random effects). Significant *p*-value is marked in bold.

Explanatory Variables	*p*	R^2^m (%)	R^2^c (%)
Treatment × DWV	**0.022**	32	72
DWV	0.203	2	76
Treatment × Varroa	0.176	24	71
Varroa	0.706	0.5	68
Treatment × Colony strength	0.475	18	72
Colony strength	0.127	<0.01	72
Treatment	0.886	17	71

## Supplementary Materials

The supplementary materials are available online at http://www.mdpi.com/2075-4450/8/1/15/s1.
